# Association between successful smoking cessation and changes in marital and job status and health behaviours: evidence from a 10-wave nationwide survey in Japan

**DOI:** 10.1186/s12889-018-5970-z

**Published:** 2018-08-22

**Authors:** Takashi Oshio

**Affiliations:** Institute of Economic Research, Hitotsubashi University, 2-1 Naka, Kunitachi, Tokyo, 186-8603 Japan

**Keywords:** Smoking cessation, Marital status, Job status, Health behaviour, Japan

## Abstract

**Background:**

There is limited knowledge the association of smoking cessation with changes in lifestyle and health behaviours. This study examined this issue using large-scale, long-term longitudinal data.

**Methods:**

The data were obtained from a 10-wave (nine-year) longitudinal nationwide survey of middle-aged individuals conducted from 2005 to 2014 in Japan. Participants included 4452 men and 1194 women aged 50–59 years who were smoking at wave 1. Smoking cessation was defined as no smoking during waves 8–10; and changes in marital and job status, leisure-time physical activity, alcohol intake, and health check-ups from waves 1 to 8 were considered. Multivariable logistic regression models were estimated to explain smoking cessation as a function of changes in marital and job status and health behaviours, and were adjusted for potential attrition bias.

**Results:**

Male smoking cessation was negatively associated with separation from a spouse (odds ratio [OR]: 0.52; 95% confidence interval [CI]: 0.29–0.92) and stopping of health check-ups (OR: 0.63; 95% CI: 0.49–0.81), while it was positively associated with moving from work to retirement (OR: 1.67; 95% CI: 1.23–2.26), beginning a leisure-time physical activity (OR: 2.37; 95% CI: 1.83–3.08), and quitting alcohol intake (OR: 1.80; 95% CI: 1.36–2.39). Female smoking cessation was negatively associated with the stoppage of health check-ups (OR: 0.31; 95% CI: 0.18–0.53) and positively associated with quitting alcohol intake (OR: 1.86; 95% CI: 1.08–3.20).

**Conclusions:**

The results underscore the association of smoking cessation with changes in marital and job status and health behaviours and imply the need for policy measures to improve health behaviours to promote smoking cessation.

## Background

Smoking continues to be a major cause of morbidity and premature mortality, and smoking cessation has been shown to have an important effect on health improvement [1, 2]. A growing number of studies have demonstrated the association between smoking cessation and marital and job status [3–7] as well as health behaviours, such as leisure-time physical activity, alcohol intake, and regular health check-ups [3, 4, 8, 9]. However, while smoking cessation has been shown to be related to changes in marital and job status—for example, it was found that the odds of smoking cessation are reduced by a broken partnership and raised by retirement [10–13]—the association between changes in health behaviours and smoking cessation has been largely understudied. Moreover, further investigation is needed to uncover the association between the long-term success (as opposed to temporary) of smoking cessation and sustained changes in job status and health behaviours, to construct effective and reliable policy measures to encourage smokers to quit smoking.

An investigation of the factors associated with successful smoking cessation is especially important for health promotion policies in Japan; in 2015, the proportion of Japanese men over the age of 15 years who smoked on a daily basis was 30.1%, much higher than the 22.9% average for 34 countries belonging to the Organisation for Economic Co-operation and Development (for women, 7.9% and 14.3%, respectively) [14]. ‘Health Japan 21,’ a 10-year project for national health promotion launched in 2013 by the Japanese Ministry of Health, Labour, and Welfare (MHLW), considers a reduction of the adult smoking rate as one of its top priorities [15].

The present study aimed to investigate the success of smoking cessation when associated with sustained changes in marital and job status and health behaviours (specifically focusing on leisure-time physical activity, alcohol intake, and health check-ups), using longitudinal data from a 10-wave (nine-year) nationwide survey of middle-aged Japanese individuals. Participants who had been smoking at wave 1 (baseline) were considered to have successfully quit smoking if they consistently reported not smoking during the final three waves of the study (waves 8–10; 7–9-year follow-up). This relatively conservative definition was selected to capture the long-term correlates of successful smoking cessation by excluding temporary (i.e., unsuccessful) smoking cessation. Participants were considered to have changed their status and behaviours only if they changed them between waves 1 and 8 and maintained the changes during the final three waves of the study.

## Methods

### Study sample

The data were obtained from a nationwide, 10-wave panel survey, the Longitudinal Survey of Middle-Aged and Older Adults, which was conducted by the MHLW each year from 2005 to 2014 for the purpose of analysing changes in the health and well-being of middle-aged and older adults. Samples in the first wave were collected nationwide from individuals between the ages of 50 and 59 years in November 2005, through a two-stage random-sampling procedure. First, 2515 districts were randomly selected from 5280 districts used in the MHLW’s nationwide, population-based Comprehensive Survey of the Living Conditions of People on Health and Welfare, which was conducted in 2004. The 5280 districts, in turn, were randomly selected from approximately 9,40,000 national census districts. Second, depending on the population size of each district, 40,877 residents, aged 50–59 years as of October 30, 2005, were randomly selected.

The questionnaires were physically distributed to the participants’ homes, where they were completed by the participants by 2 November, and physically collected several days thereafter. A total of 34,240 individuals responded (response rate: 83.8%). Waves 2–10 of the survey were conducted from 2006 to 2014. Unlike the first wave, the questionnaire was only mailed to those participants who had mailed back the questionnaire from the previous wave or the one prior to that. No new respondents were added after the first wave. The structure of the study sample is summarised in Table 1.

**Table 1:** Structure of the study sample in the panel dataset

Wave	Survey year	Number of respondents^a^	Ages of respondents	Response rate (%)	Attrition rate (%)
1	2005	34,240	50–59	83.8	–
2	2006	32,285	51–60	92.2	5.7
3	2007	30,730	52–61	95.4	4.8
4	2008	29,605	53–62	96.2	3.7
5	2009	28,736	54–63	97.3	2.9
6	2010	26,220	55–64	91.8	8.8
7	2011	25,321	56–65	90.0	3.4
8	2012	24,026	57–66	90.9	5.1
9	2013	23,722	58–67	93.9	1.3
10	2014	22,748	59–68	93.9	4.1

Note. ^a^No new respondents were added after wave 1

After excluding the participants who were missing key variables used in the statistical analyses (see below), the sample included 33,422 individuals (16,319 men and 17,103 women) who participated in wave 1. Of those participants, 10,249 individuals (30.7%), including 7996 men (49.0%) and 2253 women (13.2%), were smoking at wave 1. After excluding those who dropped out of the survey before wave 10, the final study sample included 5646 participants (4452 men and 1194 women). All of these participants reported smoking at wave 1, and some successfully quit smoking.

### Measures

#### Outcome variable: smoking cessation

**Fig. 1:**
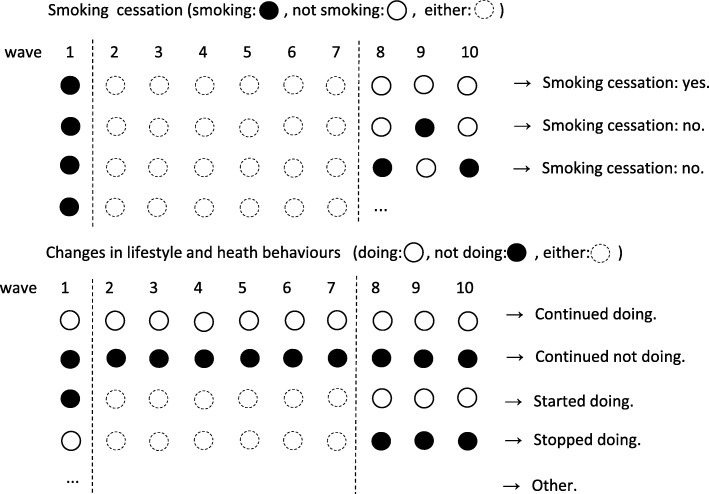
Smoking cessation and changes in marital and job status and health behaviours: their illustrative definitions

Participants who answered ‘yes’ to the question ‘do you smoke currently?’ at wave 1 were considered current smokers. At that point, a binary variable of smoking cessation was created by allocating ‘1’ to those who consistently answered ‘no’ during waves 8 and 10 and ‘0’ to those who did not. Hence, for example, those who reported smoking at least once during waves 8 and 10 were not considered as having successfully quit smoking. Figure 1 illustrates the definitions of smoking cessation changes in marital and job status and health behaviours.

#### Explanatory variables: marital and job status and health behaviours

Marital status, job status and health behaviours were each divided into five or six categories, as shown in the first columns of Tables 3 and 5 (see below in Results), and binary variables corresponding to each category were constructed. Specifically, the five binary variables of marital status that were created were ‘stayed married’, ‘stayed unmarried’, ‘got divorced/widowed’, ‘got married/remarried’ and ‘other’. The first two categories corresponded to having a spouse and no spouse, respectively, throughout the 10 waves. The following two categories indicated changes in marital status between waves 1 and 8, followed by no changes after wave 8. The remaining participants were categorised as ‘other’, which included, for example, those who changed their marital status after wave 8.

Job status was categorised into ‘continued working’, ‘continued not working’, ‘retired’, ‘stopped working (not retired)’, ‘started working’ and ‘other’. The first two meant that there was no change in job status throughout the 10 waves, and the following three indicated changes in job status between waves 1 and 8 followed by no changes after wave 8. ‘Continued working’ implied that the individual had been working before wave 1. The distinction between ‘retired’ and ‘stopped working (not retired)’ was based on whether public pension benefits were obtained or not. Note that the lowest eligible age for public pension benefits is 60–65 years, depending on the gender, birth year and occupation of the individual. ‘Other’ included the remaining participants.

Similar categorisations for changes in the three health behaviours were also made. The following binary variables were created using participants’ answers to the question about their engagement in a leisure-time physical activity in daily life: ‘continued to engage’, ‘continued not engaging’, ‘started engaging’, ‘stopped engaging’ and ‘other’. For alcohol intake, the following variables were created using participants’ answers: ‘continued drinking’, ‘continued not drinking’, ‘started drinking’, ‘stopped drinking’ and ‘other’. Finally, for health check-ups, the following variables were created using participants’ answers: ‘continued undergoing’, ‘continued not undergoing’, ‘started undergoing’, ‘stopped undergoing’ and ‘other’.

#### Control variables

Age (50–59 years), self-rated health status at wave 1 (very good, good, somewhat good, somewhat poor, poor, and very poor), and educational attainment (below high school, high school, and above high school) were included as control variables. Age was used as a continuous variable, while self-rated health and educational attainment were used as a set of binary variables for each category.

### Analytic strategy

Descriptive analyses included an examination of how the probability of smoking cessation differed across different types of changes (and no changes) in each variable of marital and job status and health behaviours, which were unadjusted for their interactions or covariates. To this end, an analysis of variance was conducted to test the null hypothesis of equal probabilities of smoking cessation.

Multivariable logistic regression models were estimated to predict smoking cessation by a set of binary variables corresponding to each change (and no change) in marital and job status and health behaviours, along with the control variables. To mitigate potential attrition biases due to dropouts, inverse probability weighting was employed [16, 17]. In general, inverse probability weighting refers to the weighting of outcome measures by the inverse of the probability of the individual with a given set of attributes assigned for their treatment. In the current study, the respondents were weighed by the inverse of the probability of their remaining in the survey throughout the 10 waves. Specifically, the probit model, which was used to predict observation presence through wave 10, was first estimated using the individual characteristics observed at baseline, including age, educational attainment, self-rated health, marital status (having a spouse or not), job status (working or otherwise), leisure-time physical activity (engaging or not engaging), alcohol intake (drinking or not drinking) and health check-ups (undergoing or not undergoing). Then, the inverse of the predicted probability of presence was used as the weight when estimating a multivariable logistic model to predict smoking cessation.

These descriptive and regression analyses were conducted separately for men and women, as previous research suggested that the determinants of smoking cessation are different for men and women [18, 19]. The statistical significance level was set at 5%.

## Results

Table 2 summarises the structure of the study sample. Among the men and women who were smoking at wave one, 31.7% (95% confidence interval (CI), 30.4–33.1%) and 30.4% (95% CI, 27.8–33.0%), respectively, quit smoking at wave 8 or before and did not smoke after that. It should be noted that 44.3% and 47.0% of men and women who were smoking at wave 1 dropped out from the survey before wave 10, underscoring the need to adjust for potential attrition biases (as discussed later). The probability of smoking relapse, which was not specifically examined in this study, was quite low: 3.8% and 1.3% for men and women, respectively.

**Table 2:** Prevalence of smoking at baseline and the final 3 waves (7 to 9-year follow-up)

	At wave 1 (baseline)		At wave 8–10 (7–9-year follow-up)			Drop-outs (C)
	Total (A = B + C)		Succeeded in quitting smoking^a^		Total (B)	[C/A]
			Yes	No		
Men	Smoking	7996	1413	3039	4452	3544
			(31.7%)	(68.3%)	(100%)	[44.3%]
	Not smoking	8323	5127	203	5330	2993
	Total	16,319	6540	3242	9782	6537
Women	Smoking	2253	363	831	1194	1059
			(30.4%)	(69.8%)	(100%)	[47.0%]
	Not smoking	14,850	9817	125	9942	4908
	Total	17,103	10,180	956	11,136	5967
Total	Smoking	10,249	1776	3870	5646	4603
			(31.5%)	(68.6%)	(100%)	[44.9%]
	Not smoking	23,173	14,944	328	15,272	7901
	Total	33,422	16,720	4198	20,918	12,504

Note. ^a^Quit smoking at wave 8 or before and did not smoke after that

Table 3 compares the probabilities of smoking cessation across changes in marital and job status and health behaviours, unadjusted for covariates. In the case of male smokers, the probability of smoking cessation was much lower among those who were divorced/widowed (20.2%), compared to those who stayed married (33.7%). Those who belonged to the groups that stayed unmarried, got married, and other lay between them. Changes in job status also affected smoking cessation; the probability of smoking cessation was the highest among those who had retired (48.8%). Regarding changes in leisure-time physical activity, beginning this kind of activity corresponded to the highest probability of smoking cessation (46.6%). As for alcohol intake, its cessation was accompanied by the highest probability of smoking cessation (43.9%). Finally, smoking cessation was less prevalent among those who stopped undergoing health check-ups (27.3%), compared to those who continued undergoing them. For all marital and job status and health behaviour variables, the null hypothesis of equal probabilities of smoking cessation across different changes and no changes was rejected at a 0.1% significance level.

**Table 3:** Probabilities of smoking cessation corresponding to changes in marital and job status and health behaviours^a^

Changes from wave 1 (baseline) to waves 8–10 (7 to 9-year follow-up)	Men		Women	
	*n*	%	*n*	%
Marital status				
Stayed married	3596	33.7	736	32.3
Got divorced/widowed	84	20.2	67	25.4
Stayed unmarried	442	23.3	254	27.2
Got married/remarried	30	23.3	18	38.9
Other	300	24.7	119	26.9
*p*-value for *F* statistics	< 0.001		0.324	
Job status				
Continued working	2853	29.7	531	29.6
Retired	205	48.8	63	41.3
Stopped working (not retired)	85	31.8	46	26.1
Stayed not working	156	28.8	234	34.2
Started working	52	28.8	34	23.5
Other	1101	34.5	286	28.0
*p*-value for *F* statistics	< 0.001		0.204	
Leisure-time physical activity				
Continued not engaging	1578	25.6	386	28.2
Started engaging	313	46.6	77	39.0
Continued engaging	108	45.4	35	45.7
Stopped engaging	304	24.0	102	28.4
Other	2149	34.5	594	30.1
*p*-value for *F* statistics	< 0.001		0.107	
Alcohol intake				
Continued drinking	2705	29.7	359	25.6
Quit drinking	230	43.9	77	39.0
Continued not drinking	789	31.3	455	33.6
Started drinking	62	32.3	31	16.1
Other	666	36.2	272	30.5
*p*-value for *F* statistics	< 0.001		0.019	
Heath check-ups				
Continued undergoing	1247	34.1	195	42.6
Stopped undergoing	447	27.3	135	20.0
Continued not undergoing	254	24.0	90	17.8
Started undergoing	220	36.4	87	36.8
Other	2284	31.7	687	29.8
*p*-value for *F* statistics	< 0.001		< 0.001	
Total	4452	31.7	1194	30.2

^a^Unadjusted for covariates

Compared to men, the differences in the probabilities of smoking cessation were more nebulous among women, based on the *p*-values of *F* statistics. Among women, the probability of smoking cessation was highest for those who quit drinking (39.0%) and relatively low for those who stopped undergoing health check-ups (20.0%). However, changes in marital status, job status, or leisure-time physical activity were not significantly associated with the probability of smoking cessation.

Table 4 compares baseline characteristics between the baseline smokers who remained in the study and those who dropped out. The drop-outs were more likely to have no spouse, lower educational attainment, poorer self-reported health (very poor or poor) among both men and women, and to have fewer chances of health check-ups among men, while there was no significant difference in physical activity or alcohol intake among both genders. These baseline differences were incorporated in the inverse probability weighting in regression models, which are discussed below to mitigate potential attrition biases.

**Table 4:** Baseline characteristics: baseline smokers who remained in the study vs. those who dropped out

	Remained	Dropped out	Difference	
	A	B	A - B	*p-*value
Men (*n* = 7996)				
Age (years, mean)	54.7	54.4	0.2	< 0.001
Married (%)	88.3	81.0	7.4	< 0.001
Graduated from high school or below (%)	74.2	65.6	8.6	< 0.001
Poor self-rated health^a^ (%)	3.5	6.2	−2.7	< 0.001
Physical activity (%)	19.1	20.7	−1.6	0.082
Alcohol drinking (%)	75.4	74.5	0.9	0.340
Heath check-up (%)	77.7	70.3	7.3	< 0.001
*n*	4452	3544		
Women (*n* = 2253)				
Age (years, mean)	54.4	54.4	0.0	0.913
Married (%)	74.3	71.4	2.9	0.122
Graduated from high school or below (%)	83.0	71.1	11.9	< 0.001
Poor self-rated health (%)	4.3	7.5	−3.2	0.001
Physical activity (%)	23.7	25.7	−2.0	0.276
Alcohol drinking (%)	46.4	47.5	−1.1	0.602
Heath check-up (%)	64.3	57.3	7.1	< 0.001
*n*	1194	1059		

^a^Included very poor and ‘poor

Table 5 summarises the results of the multivariable logistic regression models used to explain smoking cessation by changes (and no changes) in marital and job status and health behaviours, adjusted for age, self-rated health at wave 1, educational attainment, and potential attrition biases. The reference categories were ‘stayed married’, ‘continued working’, ‘continued not engaging in leisure-time physical activity’, ‘continued drinking’ and ‘continued undergoing health check-ups’, which had the largest proportions at baseline for each marital and job status and health behaviours variable. Estimation results were largely consistent with those reported in Table 2.

**Table 5:** Estimated associations of smoking cessation with changes in marital and job status and health behaviours^a^

Changes from wave 1 (baseline) to waves 8–10 (7 to 9-year follow-up)	Men (*N* = 4452)		Women (*N* = 1194)	
	OR	95% CI	OR	95% CI
Marital status				
Stayed married	1		1	
Got divorced/widowed	0.52^*^	0.29–0.92	0.72	0.40–1.31
Stayed unmarried	0.63^***^	0.49–0.81	0.84	0.60–1.19
Got married/remarried	0.67	0.27–1.66	1.27	0.50–3.22
Other	0.62^***^	0.46–0.83	0.83	0.52–1.33
Job status				
Continued working	1		1	
Retired	1.67^***^	1.23–2.26	1.62	0.92–2.87
Stopped working (not retired)	1.08	0.65–1.79	0.88	0.43–1.81
Stayed not working	0.98	0.66–1.46	1.25	0.87–1.81
Started working	0.98	0.53–1.84	0.85	0.38–1.93
Other	1.12	0.95–1.32	0.96	0.68–1.35
Leisure-time physical activity				
Continued not engaging	1		1	
Started engaging	2.37^***^	1.83–3.08	1.51	0.87–2.61
Continued engaging	2.21^***^	1.48–3.30	1.54	0.75–3.16
Stopped engaging	0.95	0.71–1.27	0.93	0.55–1.55
Other	1.50^***^	1.29–1.74	1.05	0.78–1.42
Alcohol intake				
Continued drinking	1		1	
Quit drinking	1.80^***^	1.36–2.39	1.86^*^	1.08–3.20
Continued not drinking	1.16	0.96–1.39	1.39^*^	1.01–1.93
Started drinking	1.22	0.68–2.21	0.53	0.18–1.52
Other	1.43^***^	1.19–1.72	1.19	0.81–1.73
Heath check-ups				
Continued undergoing	1		1	
Stopped undergoing	0.63^***^	0.49–0.81	0.31^***^	0.18–0.53
Continued not undergoing	0.71^*^	0.51–0.99	0.25^***^	0.13–0.47
Started undergoing	1.23	0.90–1.68	0.76	0.44–1.31
Other	0.88	0.75–1.03	0.56^***^	0.39–0.78

^a^Adjusted for ages and self-rated health at baseline, educational attainment, and attrition biases

^***^p < 0.001, ^*^p < 0.05

As shown in Table 3, male smoking cessation was negatively associated with separating from a spouse (OR = 0.52; 95% CI, 0.29–0.92) and stopping health check-ups (OR = 0.63; 95% CI, 0.49–0.81), whereas it was positively associated with retiring (OR = 1.67; 95% CI, 1.23–2.26), starting a leisure-time physical activity (OR = 2.37; 95% CI, 1.83–3.08), and quitting alcohol intake (OR = 1.80; 95% CI, 1.36–2.39). The estimated ORs were well below 1 for the negative associations and well above 1 for positive associations.

Compared to male smokers, the associations of smoking cessation with changes in marital and job status and health behaviours were more nebulous among female smokers. Female smoking cessation was negatively associated with stopping health check-ups (OR = 0.31, 95% CI, 0.18–0.53) and positively associated with quitting alcohol intake (OR = 1.86; 95% CI, 1.08–3.20). However, smoking cessation was not associated with changes in other variables among women, at least partly because of the small sample size in the corresponding category of the explanatory variable (see Table 3).

Owing to space limitations, the estimation results of the probit model, which explained observation presence through wave 10 and calculated the probability of presence used for inverse probability weighting, are not presented here (available upon request). The results indicated that observation presence was higher with higher educational attainment, better self-rated health, and more health check-ups for both men and women, in line with the results presented in Table 3.

Additionally, the multivariable logistic regression models, which included the binary variable of women and its interaction terms with key variables, were estimated. The results, which are not presented to conserve space but are available upon request, were generally consistent with those in Table 5. The ORs of the interaction terms with ‘continued undergoing’ and ‘continued not undergoing’ health check-ups were significantly below 1, indicating a closer association between health check-ups and smoking cessation among women. In contrast, the ORs of the interaction terms of other variables were non-significant, probably reflecting (i) their non-significant associations with smoking cessation among women (for marital status, job status, and leisure-time physical activity), or (ii) little difference in the association between men and women (for alcohol intake).

## Discussion

This study examined the association between smoking cessation and changes in marital and job status and health behaviours using data from a 10-wave longitudinal nationwide survey of middle-aged Japanese individuals. The results confirmed that smoking cessation was closely associated with changes in marital and job status and health behaviours, especially for men. Among male smokers, smoking cessation was negatively associated with separating from a spouse and stopping health check-ups and positively associated with retiring, starting a leisure-time physical activity, and quitting alcohol intake. Smoking cessation among females was less sensitive to changes in marital and job status and health behaviours, but was positively associated with alcohol cessation and negatively associated with stopping regular health check-ups, similar to male smokers.

These results were generally consistent with the results obtained from previous studies that addressed the predictors of successful smoking cessation or smoking behaviours in general [3–13]. However, the novelty of the current study was that it uncovered the associations of smoking cessation with sustained changes in its potential predictors, which provided new insights into their relevance for smoking behaviours. The results also suggest that sustained changes in marital and job status and health behaviours may be irreversibly associated with individuals’ attitudes towards health, leading to sustained changes in smoking behaviour.

Gender differences in the association between smoking cessation and changes in marital and job status and health behaviours were also observed. While quitting drinking and stopping health check-ups were closely related to smoking cessation in both men and women, changes in marital status, job status, or leisure-time physical activity were not related to female smoking cessation. Exploring the determinants of these gender differences was beyond the scope of the current study. However, it is possible that the relatively limited sensitivity of female smoking cessation to changes in marital and job status and health behaviours may be related to socio-cultural backgrounds. Previous studies or official statistics in Japan have observed that (i) the subjective well-being of women tends to be less sensitive to marital status [20], (ii) full-time jobs are less prevalent among middle-aged women (55.1% compared to 91.4% for men [aged 55–64 years in 2016]) [21], and (iii) the combination of work and family life is more diversified for women [22]. The socio-cultural backgrounds reflected in these observations may at least partly account for the non-significant associations of female smoking cessation with changes in marital and job status and leisure-time physical activity.

Despite these gender differences, the results suggest that policy support can be constructed to encourage smokers to quit smoking. Notably, policy measures to encourage individuals to keep undergoing health check-ups are expected to maintain the pace of smoking cessation. In addition, policy measures intended to encourage individuals to engage in a leisure-time physical activity may be effective in increasing the probability of smoking cessation. For male smokers, these policy measures are expected to be most effective if they are targeted towards those in transition from working life to retirement, because they are likely to lose opportunities to attend company-sponsored health check-ups (which are required by law in Japan) after they retire and also because they tend to become more inclined to quit smoking after retirement, as observed in this study.

This study had several limitations and drawbacks. First, it was not possible to identify any clear causation from changes in marital and job status or health behaviours related to smoking cessation, because changes in marital and job status or health behaviours and smoking cessation occurred simultaneously for some respondents and also because the possibility of their interactions during waves 2 and 7 could not be excluded. Second, there could be other factors correlated with smoking cessation that were not analysed in this study, including psychological distress [23], social support [24] and financial stress [25]. Third, the study sample was limited to middle-aged Japanese individuals and was substantially male-dominated, thus requiring the need for caution in making any generalisation of the results.

However, the observations in this study are reliable and robust for two reasons. First, smoking cessation and changes in marital and job status and health behaviours were defined in relatively conservative ways, which should prevent the distortion of the results because of temporary changes. Second, potential attrition biases were mitigated by applying inverse probability weighting, which is a well-established method in econometrics, assuming that the attrition depended on the participants’ attributes which were observed at baseline or were time-invariant.

## Conclusions

In summary, this study underscores the association of successful smoking cessation with changes in marital and job status and health behaviours among middle-aged smokers, especially men. The results imply that policy measures to improve health behaviours can help encourage people to quit smoking.

## Abbreviation

MHLW: Ministry of Health, Labour and Welfare
